# Red blood cell complement receptor one level varies with Knops blood group, α^+^thalassaemia and age among Kenyan children

**DOI:** 10.1038/gene.2016.2

**Published:** 2016-02-04

**Authors:** D H Opi, S Uyoga, E N Orori, T N Williams, J A Rowe

**Affiliations:** 1 Kenya Medical Research Institute-Wellcome Trust Research Programme, Kilifi, Kenya; 2 Centre for Immunity, Infection and Evolution, Institute of Immunology and Infection Research, School of Biological Sciences, University of Edinburgh, Edinburgh, UK; 3 Department of Medicine, Imperial College, St Mary's Hospital, London, UK

## Abstract

Both the invasion of red blood cells (RBCs) by *Plasmodium falciparum* parasites and the sequestration of parasite-infected RBCs in the microvasculature are mediated in part by complement receptor one (CR1). RBC surface CR1 level can vary between individuals by more than 20-fold and may be associated with the risk of severe malaria. The factors that influence RBC CR1 level variation are poorly understood, particularly in African populations. We studied 3535 child residents of a malaria-endemic region of coastal Kenya and report, for the first time, that the CR1 Knops blood group alleles *Sl2* and *McC*^*b*^, and homozygous HbSS are positively associated with RBC CR1 level. Sickle cell trait and ABO blood group did not influence RBC CR1 level. We also confirm the previous observation that α^+^thalassaemia is associated with reduced RBC CR1 level, possibly due to small RBC volume, and that age-related changes in RBC CR1 expression occur throughout childhood. RBC CR1 level in malaria-endemic African populations is a complex phenotype influenced by multiple factors that should be taken into account in the design and interpretation of future studies on CR1 and malaria susceptibility.

## Introduction

*Plasmodium falciparum* is the most common cause of malaria world wide and is responsible for the vast majority of malaria deaths.^1^ *P. falciparum* exploits multiple host receptors during the process of red blood cell (RBC) invasion and in the subsequent sequestration of *P. falciparum* parasitised RBCs in the microvasculature.^2^ Understanding these host–parasite receptor–ligand interactions may help in the development of new methods for preventing and treating malaria.^2^ Complement receptor one (CR1) is a single-chain *trans*-membrane glycoprotein that is expressed on the surface of RBCs and leukocytes. The normal function of RBC CR1 is to regulate complement activation and to transfer immune complexes to phagocytic cells in the liver and spleen.^3^ In malaria, CR1 acts as a receptor on RBCs for both *P. falciparum* invasion^4, 5, 6, 7^ and rosette formation, in which two or more uninfected RBCs bind to a *P. falciparum* parasitised RBC *in vitro*.^8, 9^ Rosetting has been associated with severe malaria in multiple African study sites (reviewed in Rowe *et al.*^10^), potentially because it leads to enhanced parasitised RBC sequestration and decreased blood flow in post-capillary venules.^11, 12^ CR1 acts as a receptor for *P. falciparum* RBC invasion via its interaction with the parasite ligand *P. falciparum* reticulocyte-binding-like homologue protein 4.^4, 5, 6, 7^

The mean number of CR1 molecules expressed on the RBC surface varies between individuals, typically between 50 and 1500 CR1 molecules/RBC. RBC with <200 CR1 molecules per cell show reduced rosetting^8^ and negative or weak reactions with CR1 antibodies,^13^ being most marked at <100 CR1 molecules per cell. Low RBC CR1 level has also been associated with reduced *P. falciparum* RBC invasion *in vitro*.^5, 6^ Individuals with low RBC CR1 are common in some malaria-endemic regions,^14, 15^ with up to 80% of the population in coastal Papua New Guinea having fewer than 200 CR1 molecules per RBC.^14^ Variation in RBC CR1 level may contribute to individual differences in susceptibility to severe malaria. Low RBC CR1 might protect against cerebral malaria through the mechanism of reduced rosetting,^8, 9, 14^ whereas, in contrast, increasing the risk of immune-complex-mediated tissue damage and severe malarial anaemia.^16, 17^

Factors determining the variation in RBC CR1 expression are not fully understood, but are proposed to be both genetic and acquired.^18^ In Caucasian and Asian populations, variation in RBC CR1 level is genetically determined and is associated with a *Hin*dIII restriction fragment length polymorphism that is in tight linkage disequilibrium with several other single-nucleotide polymorphisms (SNPs) in exons 19, 22 and 33 of the *CR1* gene.^19, 20, 21, 22^ However, in African populations, although RBC CR1 levels vary over the same range, there is no association between CR1 level and any of the above SNPs, and the causal mutations that determine RBC CR1 levels are unknown.^21, 23^ *CR1* is also the site for SNPs giving rise to the Knops blood group system of antigens that include the allelic pairs Swain-Langley 1 and 2 (*Sl1* and *Sl2*), McCoy a and b (*McC*^*a*^ and *McC*^*b*^) and Knops a and b (*Kn*^*a*^ and *Kn*^*b*^).^24, 25, 26, 27^ *Sl2* and *McC*^*b*^ occur at higher frequencies in African populations than non-African populations.^28^ To the best of our knowledge, the effect of the Knops *Sl* and *McC* genotypes on RBC CR1 level has not been studied previously.

One study in Papua New Guinea found a dose-dependent decrease in RBC CR1 level with α^+^thalassaemia,^14^ an association that might exist in African populations where α^+^thalassaemia is also common. It is unknown whether other high-frequency African polymorphisms associated with malaria resistance such as HbS and blood group O might influence RBC CR1 level. Two previous studies have shown that RBC CR1 expression level varies with age in both malaria-endemic and non-endemic populations, being lowest during early childhood.^16, 29^

In the current study, we investigated RBC CR1 level and the factors that influence it in children living within a malaria-endemic region on the Kenyan coast.

## Results

### RBC CR1 level in the Kilifi population varies with age during childhood

**Figure 1:**
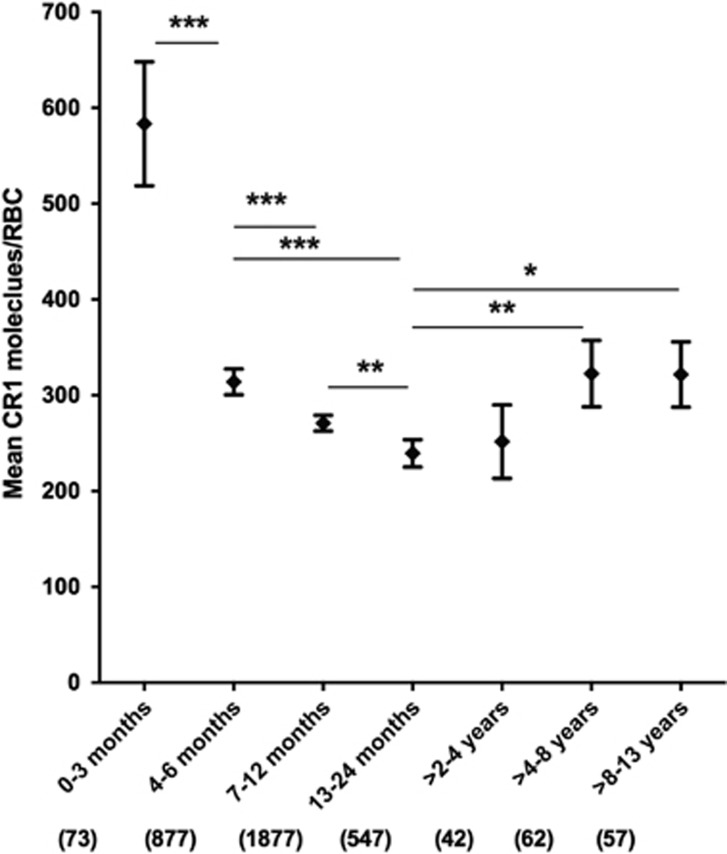
RBC CR1 level varies with age. The relationship between RBC CR1 level and age was examined in a total of 3535 individuals aged between 0 months and 13 years by one-way analysis of variance with Tukey's *post-hoc* test to correct for multiple comparisons. Age was presented as a categorical variable defined into seven categories: 0–3 months, 4–6 months, 7–12 months, 1–2 years, >2–4 years, >4–8 years and >8–13 years. Error bars represent 95% confidence intervals of the mean RBC CR1 levels. Numbers in parenthesis reflect total number of samples tested for each group. Statistically significant differences in RBC CR1 level between groups are asterisked, *P<0.05, **P<0.01 and ***P<0.001. All age groups differed significantly from the 0–3 months age group (*P* <0.001), but these are not all asterisked on the graph in order to enhance readability of the figure.

We measured RBC CR1 level by flow cytometry in a total of 3535 children 0–13 years of age in Kilifi County on the coast of Kenya. Previous work in Western Kenya suggested that RBC CR1 levels change with age during childhood,^16, 29^ and this relationship was confirmed in the Kilifi population (Figure 1). We analysed the data by analysis of variance using the same age categories as previous studies,^16, 29^ and found that mean RBC CR1 level was higher in newborns (0–3 months) than in other age groups, and significantly declined during each age interval in the first two years of life (Figure 1). CR1 level then increased and stabilized in older children (>4 years). We also analysed the relationship between RBC CR1 level and age as a continuous variable, using piecewise-linear regression analysis, which allows for adjustment for known confounding variables such as α^+^thalassaemia.^14^ This analysis assumes a linear effect within defined age ranges, using the same age categories as Figure 1. This confirmed the significant decline in RBC CR1 levels during each age category up to 2 years (shown by negative regression coefficients in Table 1). Then between 2 and 4 years of age, the RBC CR1 level significantly increased with age (shown by a positive regression coefficient, Table 1). There was no statistically significant change in CR1 level in the >4–8 years and >8–13 years age intervals, showing that CR1 levels plateau by about 4 years of age, as suggested previously.^16, 29^ When analysed across all samples, the regression analysis gave a value of *r*^2^=0.05, indicating that although there are significant changes in CR1 level within defined age groups as indicated above, overall, age explains only a minor part of the variation in RBC CR1 level, and other factors must be playing a role.

### RBC CR1 level is negatively associated with α^+^thalassaemia genotype

**Figure 2:**
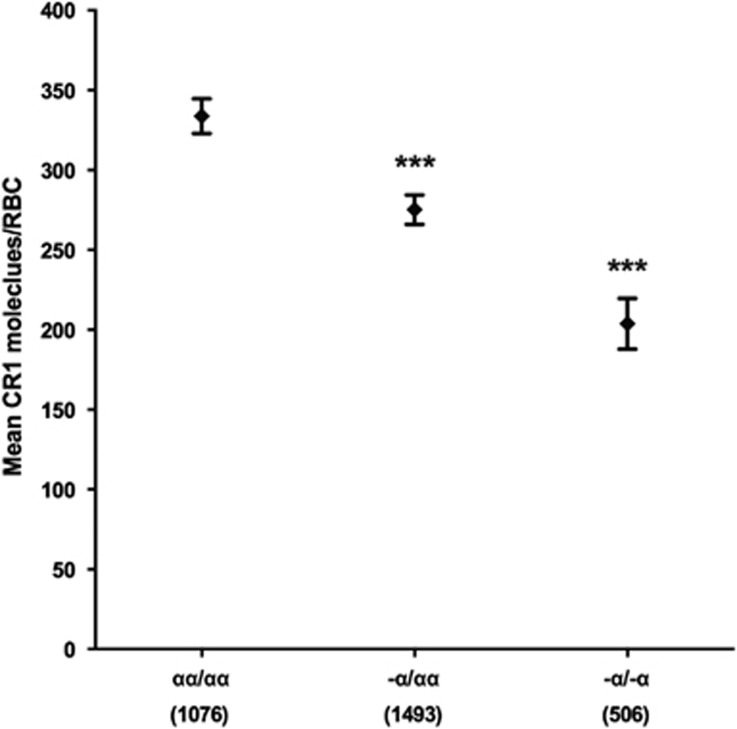
RBC CR1 level is negatively associated with α^+^thalassaemia genotype. RBC CR1 level was examined in a total of 3535 children with known α^+^thalassaemia genotypes. Differences in RBC CR1 level by α^+^thalassaemia genotype were compared by multivariate linear regression analysis with adjustment for age, HbS, *Sl* and *McC* genotypes. The normal globin genotype (αα/αα) was used as the reference genotype. Error bars represent 95% confidence intervals of the mean RBC CR1 level. Numbers in parenthesis reflect total number of samples tested for each genotype group. Statistically significant differences in RBC CR1 level from the reference genotype are asterisked, ***P<0.001.

We investigated whether the association between α^+^thalassaemia and RBC CR1 level previously reported in Papua New Guinea^14^ might also hold in the Kilifi population. Compared with normal cells (mean CR1 molecules per RBC 334; 95% confidence interval (CI)=323–345), both heterozygous and homozygous α^+^thalassaemia were associated with significantly lower RBC CR1 levels: (275; 266–284; *P*<0.001) and (204; 188–220; *P*<0.001), respectively (Figure 2).

### MCV largely accounts for the association between α^+^thalassaemia and RBC CR1 level

**Figure 3:**
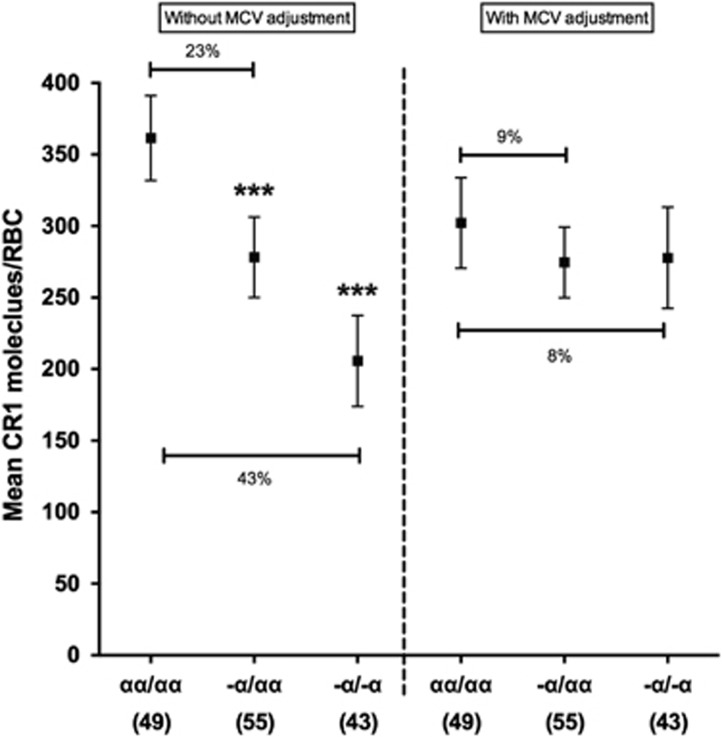
MCV largely accounts for the association between α^+^thalassaemia and RBC CR1 level. Association between RBC CR1 level and α^+^thalassaemia in 147 samples without adjustment for MCV (left side), or with adjustment for MCV (right side). Differences in CR1 level by α^+^thalassaemia were first analysed by multivariate linear regression analysis with adjustment for age, HbAS and *Sl* and *McC* genotypes and compared with a separate model with the additional adjustment for MCV. The normal globin genotype (αα/αα) was used as the reference genotype. Error bars represent 95% confidence intervals of the mean RBC CR1 level. The percentages shown represent the relative difference in mean CR1 level between the respective genotypes. Numbers in parenthesis reflect total number of samples tested for each genotype group. Statistically significant differences in RBC CR1 level from the reference genotype are asterisked, ***P<0.001.

The mechanism leading to reduced RBC CR1 level with α^+^thalassaemia has not been explored previously. α^+^Thalassaemia RBCs are microcytic, with significantly lower mean cell volumes (MCVs) compared with normal cells. We therefore examined whether reduced MCV could explain the lower CR1 levels seen with α^+^thalassaemia. One hundred and forty-seven RBC samples from the Mild Disease Cohort included in the above analyses had MCV measurements available. As expected, α^+^thalassaemia was associated with reduced MCV in both the heterozygous (MCV=73.5; 95% CI=72.0–74.9; *P*<0.001) and homozygous state (65.1; 63.5–66.8; *P*<0.001) when compared with normal RBCs (79.6; 78.0–81.1). As seen in the larger data set, unadjusted for MCV, RBC CR1 levels declined with α^+^thalassaemia in the 147 samples: CR1 level in heterozygous (278; 250–306, *P*<0.001) and homozygous (206; 174–237; *P*<0.001) α^+^thalassaemic RBCs compared with normal RBCs (361; 332–391; Figure 3, left side). After adjusting for MCV, the difference in CR1 level between normal RBCs (302; 271–334) and heterozygous (275; 250–299, *P*=0.172) and homozygous α^+^thalassaemic RBCs (278; 242–313, *P*=0.382) was no longer statistically significant (Figure 3, right side).

### RBC CR1 level is affected by homozygous sickle cell disease (HbSS) but not by heterozygous sickle cell trait (HbAS) or ABO blood group

**Figure 4:**
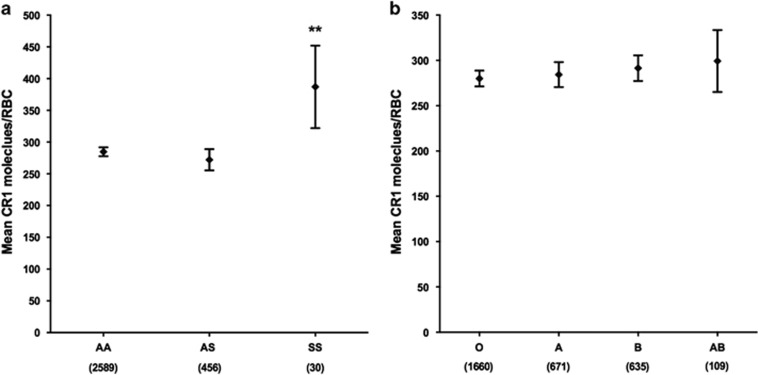
RBC CR1 level increases with homozygous HbS (HbSS) but is not affected by sickle cell trait (HbAS) or ABO blood group. (**a**) RBC CR1 level by HbS genotype. (**b**) RBC CR1 level by ABO blood group. Differences in RBC CR1 level were examined in a total of 3075 individuals by multivariate regression analysis with adjustment for age, α^+^thalassaemia, *Sl* and *McC* genotypes. The normal globin genotype (HbAA) and blood group O were used as the reference groups, respectively. Error bars represent 95% confidence intervals of the mean RBC CR1 level. Numbers in parenthesis reflect total number of samples tested for each genotype group. Statistically significant differences in RBC CR1 level from the reference groups are asterisked, **P<0.01.

We also investigated the relationship between RBC CR1 level and other common polymorphisms in the Kilifi population, including the sickle cell mutation (HbS)^30^ and blood group O of the ABO blood group system^31^ in 3075 individuals for whom full genotype data were available. We found no significant association between RBC CR1 level and either sickle cell trait (HbAS; Figure 4a) or ABO blood group (Figure 4b). Although the number of subjects studied was small (*n*=30), homozygous sickle cell disease (HbSS) was associated with significantly elevated RBC CR1 level (387; 322–452; *P*=0.002) in comparison to normal HbAA RBCs (285; 278–292; Figure 4a).

### RBC CR1 level is positively associated with both the *Sl2* and *McC*^*b*^ CR1 Knops blood group genotypes

**Figure 5:**
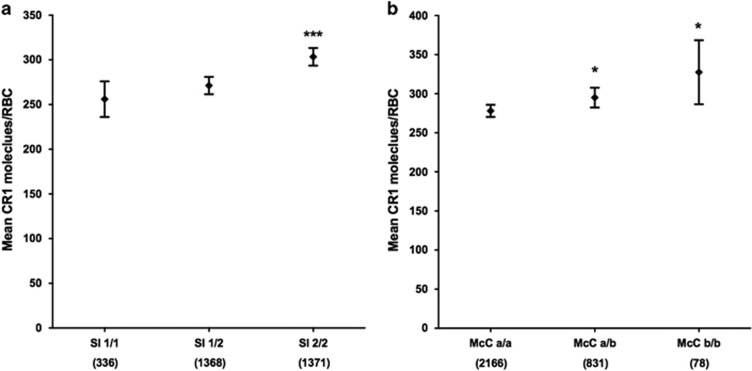
RBC CR1 level varies by Swain-Langley and McCoy genotypes. RBC CR1 level by CR1 Knops blood genotype. (**a)** Swain-Langley (*Sl*) and (**b**) McCoy (*McC*). Differences in RBC CR1 level by *Sl* and *McC* genotypes were tested in a total of 3075 individuals by multivariate linear regression analysis with adjustment for age, α^+^thalassaemia and HbS genotypes. Error bars represent 95% confidence intervals of the mean RBC CR1 level. Numbers in parenthesis reflect total number of samples tested for each genotype group. The *Sl1/1* and *McCa/a* genotypes were used as the reference genotypes, respectively. Statistically significant differences in RBC CR1 level from the reference genotypes are asterisked, *P<0.05, ***P<0.001.

**Figure 6:**
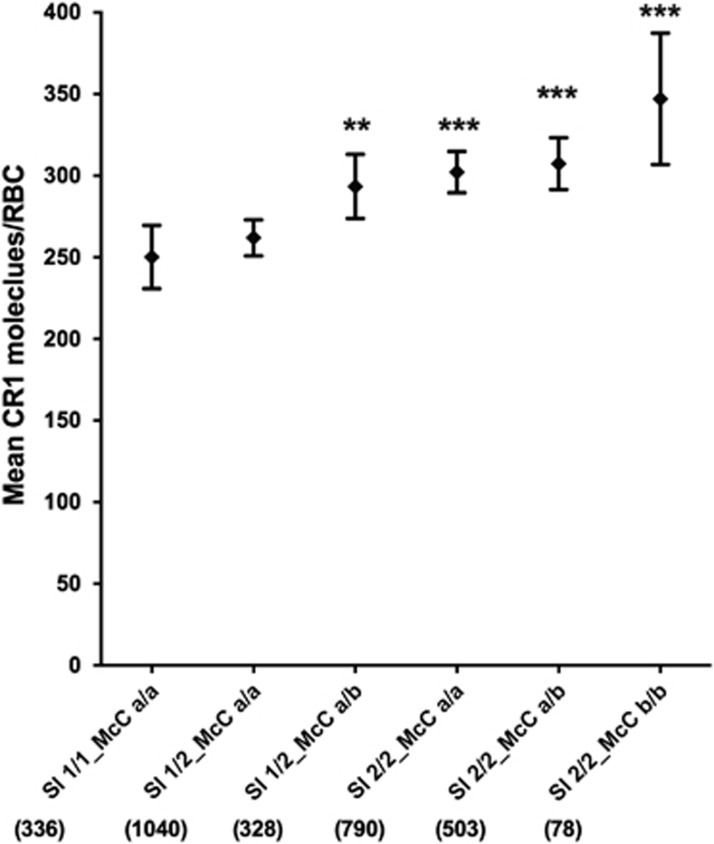
RBC CR1 level by Swain-Langley and McCoy genotype combinations. Differences in RBC CR1 level by *Sl* and *McC* genotype combinations were compared by multivariate linear regression analysis in a total of 3075 individuals with adjustment for age, α^+^thalassaemia and HbS genotypes. Error bars represent 95% confidence intervals of the mean RBC CR1 level. Numbers in parenthesis reflect total number of samples tested for each genotype combination group. Statistically significant differences in RBC CR1 level from the reference *Sl1/1*_*McCa/a* genotype combination are asterisked, **P<0.01, ***P<0.001.

The CR1 Knops blood group *Sl2* and *McC*^*b*^ alleles are present at high frequencies in African populations,^28^ but it is unknown whether they influence RBC CR1 level. We found that homozygous *Sl2* genotype was associated with an increased RBC CR1 level (303; 294–313; *P*<0.001 when compared with homozygous *Sl1* 256; 236–276; Figure 5a). Similarly, both heterozygous and homozygous *McC*^*b*^ were associated with increased RBC CR1 level (295; 282–308; *P*=0.028) and (328; 287–369; *P*=0.021), respectively, in comparison to homozygous *McC*^*a*^ (278; 270–286; Figure 5b). This association was even more marked when looking at *Sl-McC* genotype combinations, with the homozygous *Sl2-McC*^*b*^ haplotype expressing the highest CR1 level (347; 307–387; *P*<0.001), when compared with the homozygous *Sl1-McC*^*a*^ (250; 231–270; Figure 6).

## Discussion

An increasing body of evidence from *in vitro* studies supports a role for CR1 in the pathogenesis of severe malaria,^4, 5, 6, 7, 8, 9^ yet little is known about the determinants of RBC CR1 level variation in African populations. We and others have shown previously that the SNPs in the *CR1* gene that are associated with RBC CR1 level in Caucasian and Asian populations do not influence RBC level in African populations.^21, 23^ In the current study, we have shown that the CR1 Knops blood group *Sl2* and *McC*^*b*^ genotypes are associated with increased RBC CR1 levels. In addition, we have confirmed age-associated changes in RBC CR1 during childhood, and that α^+^thalassaemia is associated with reduced RBC CR1 levels, an observation that could largely be explained by the microcytic nature of thalassaemic RBCs.

To the best of our knowledge, the association between RBC CR1 level and the CR1 Knops blood group antigens has not been investigated previously. We have shown that both *Sl2* and *McC*^*b*^ are associated with increased RBC CR1 level, even after adjusting for known confounders (Figures 5 and 6). The mechanisms involved, however, remain unclear. *Sl2* and *McC*^*b*^ alleles are the products of substitution of the basic amino acid arginine for the neutral glycine and substitution of the basic amino acid lysine for the acidic glutamic acid, respectively.^27^ CR1 is known to be cleaved from the RBC surface as cells age,^32^ although the specific proteases involved are unknown. Several proteases attack peptide bonds at the amino or carboxyl side of arginine or lysine residues, therefore one hypothesis is that the *Sl2* and *McC*^*b*^ substitutions cause loss of proteolytic cleavage sites and hence result in higher CR1 levels on RBC. Alternatively, the substitutions might cause conformational changes in CR1 that might render the molecule less susceptible to proteolytic cleavage. However, previous work using nuclear magnetic resonance was unable to demonstrate any structural differences between the different polymorphic forms of CR1.^33^

The mechanism by which α^+^thalassaemia results in lower RBC CR1 levels, as shown in this study (Figure 2) and reported previously,^14^ has not yet been defined. We found that RBC size is an important factor, as the difference between thalassaemic and normal cells was no longer statistically significant after adjusting for the reduced MCV of α^+^thalassaemic RBCs^34^ (Figure 3). Microcytic RBCs due to α^+^thalassaemia and other causes such as iron-deficiency have been shown to have reduced rosette frequencies with *P. falciparum*-infected RBC, which could influence susceptibility to severe malaria.^35^ It remains unclear whether microcytic RBC synthesise lower amounts of CR1 than normal cells, or whether some CR1 is lost, for example, through exocytosis of membrane vesicles during normal RBC ageing,^36^ as well as by proteolysis as described above. Increased vesiculation has been documented in α^+^thalassaemia RBCs,^37, 38, 39^ which might therefore provide a mechanism for CR1 loss.

In agreement with two previous studies,^16, 29^ we found that RBC CR1 levels were influenced by host age in the Kilifi population (Figure 1). The mechanisms leading to these age-related changes are unknown. RBC MCV is known to vary with age, falling in early childhood up to about 15 months of age, and then steadily rising into adulthood.^40^ Changes in MCV with age were relatively minor in our data set of 147 samples (Supplementary Figure S1), and seem unlikely to account for the differences in CR1 levels between different age groups during early childhood. However, further studies of RBC CR1 level, MCV and age with larger sample sizes and over a wider age range will be needed to examine this relationship more fully.

Although factors affecting RBC CR1 level have been clearly demonstrated in this study, the work has some limitations. In addition to the Knops blood groups polymorphisms, CR1 also displays four allelic size variants: CR1-A (220 kDa), CR1-B (250 kDa), CR1-C (190 kDa) and CR1-D (280 kDa). These differences are a result of unequal crossing-over leading to duplications or deletions of a long homologous repeat (LHR) unit, with CR1-A, B, C and D consisting of 4, 5, 3 and 6 LHR's, respectively. CR1-A and B are the most common, occurring at frequencies of between 75–98% and 2–13%, respectively, whereas CR1-C and D are rare.^41^ The association between CR1 allelic variants and RBC CR1 level and Knops antigen type is unknown and was not examined in our study. Different anti-CR1 monoclonal antibodies are known to recognise epitopes on different LHRs;^42, 43^ for example, J3D3 binds epitopes on LHR-A, B and C.^43^ Therefore, depending on the overall frequencies of these different allelic variants in our population, and how they associate with the different genotypes tested, it is possible that we may have either under- or over-estimated CR1 levels using our current methods.^44^ Furthermore, the allelic types of the samples of known CR1 level used as standards in our CR1 flow cytometry assay are unknown, and again could influence the CR1 levels measured.

Another uncertainty is whether the variation in RBC CR1 level that we describe here has functional significance in terms of malaria host–parasite interactions. Previous work on CR1-mediated invasion of RBC by *P. falciparum* shows a significant positive correlation between RBC CR1 level and binding of the parasite invasion ligand *P. falciparum* reticulocyte-binding-like homologue protein 4^(ref. 7)^ and parasite invasion rate,^5^ suggesting that relatively small increases in RBC CR1 level may have the potential to increase invasion efficiency and increase overall parasite burden. Previous work on *P. falciparum* rosetting suggests that very low levels of RBC CR1 (less than 100 molecules per cell) reduce rosetting,^8, 9^ but there is no evidence for a direct positive correlation between increasing amounts of CR1 and increasing rosetting. Instead, it seems more likely that there is a threshold effect, such that cells with less than 100–200 molecules per cell show reduced rosetting, whereas those with more than 100–200 molecules per cell show similar rosetting capacity over the full range of CR1 levels (200–1500 molecules per cell). The overall levels of CR1 measured in this study are relatively low, with mean values between 250 and 350 molecules per RBC for most age groups and genotypes (Figures 1,2,3,4,5,6), but they are higher than those measured in other parts of the world where low CR1 has been associated with protection against severe malaria, such as Papua New Guinea^14^ and India.^15^ Further work will be needed to study the relationship between RBC CR1 level, *P. falciparum* invasion and rosetting and severe malaria in African populations to determine the functional significance of the CR1 level variation described here.

One unexplained contradiction in our data is that low CR1 is thought to protect against severe malaria,^14, 15^ yet the Knops mutations are associated with increased CR1 levels (Figures 5 and 6). This appears inconsistent with suggestions that the Knops mutations have been selected to high frequencies in African populations because of a protective advantage against severe malaria.^8, 45^ Recent *in vitro* data using truncated recombinant proteins with the Knops polymorphisms found no effect of the *Sl2* and *McCb* mutations on CR1 complement regulatory functions or *P. falciparum* rosetting or invasion.^33^ In addition, the SNPs determining Sl and McC blood group antigens are located within LHR-D of CR1,^8, 27^ whereas the sites implicated in rosetting and invasion are in LHR-A, -B and -C.^8, 9, 6, 46^ It is possible that the Knops polymorphisms might protect against severe malaria through mechanisms in which increased CR1 is advantageous. CR1 is involved in clearance of C3b/C4b-coated immune complexes and regulation of complement activation, and RBCs with low CR1 may be more susceptible to complement-mediated damage and increased clearance, which are mechanisms associated with severe malarial anaemia.^29, 47, 48^ Alternatively, *Sl2* and *McC*^*b*^ could be under selection through other diseases unrelated to malaria. CR1 is a receptor-mediating phagocytic uptake and cell invasion by a number of infectious agents including *Mycobacterium tuberculosis, M. leprae* and *Leishmania major*.^49, 50, 51, 52, 53, 54, 55, 56, 57^ Preliminary studies suggest that *McC*^*b*^ is associated with protection against *M. tuberculosis* and *M. leprae* in West Africa.^58, 59^ Recent evidence from our group also points to both *McC*^*b*^ and *Sl2* protecting against common non-malaria-related illnesses among children living on the Coast of Kenya (Opi, *et al.*, manuscript in preparation).

In summary, we have shown that a number of host factors including Knops blood group genotype, α^+^thalassaemia and age are associated with RBC CR1 level in Kenyan children. The exact mechanisms by which levels of RBC CR1 are controlled remain unknown, but variation in RBC CR1 might be partly explained by differences in haematological indices such as MCV or by other complex processes including susceptibility to proteases and RBC vesiculation that are potential areas for future study.

## Materials and methods

### Study populations

All samples used in this study were collected from residents of the area served by the Kilifi Health and Demographic Surveillance System (KHDSS) on the coast of Kenya. This area and the resident population have been described in detail previously.^60^ The study involved a total of 3535 individuals aged 0 months to 13 years of age recruited into three different study cohorts, each of which have been previously described: the Kilifi Genetic Birth Cohort (2928 individuals),^61^ Birth Cohort Study 2 (460 individuals)^62, 63^ and Mild Disease Cohort (147 individuals).^64, 65^ Following informed consent from parents or guardians, blood samples were collected by either venipuncture or by capillary sampling into sterile tubes containing EDTA (BD Vacutainer systems, Franklin Lakes, NJ, USA). Because CR1 levels are known to decline during malaria infections,^16^ samples were only collected from malaria negative individuals, as tested by a rapid diagnostic test (OptiMAL Diamed, Morat, Switzerland) and by light microscopy of thick and thin Giemsa-stained blood smears.

### RBC surface CR1 quantification by flow cytometry

Whole blood was washed thrice in supplemented RPMI-1640 medium (Invitrogen, Carlsbad, CA, USA; with 25 mM HEPES, 2 mM  l-glutamine (Invitrogen), 25 μg ml^−1^ gentamicin, 20 mM d-glucose (Sigma) and 6 mM NaOH) by centrifugation and plasma and white blood cells removed from the RBC pellet by aspiration. RBC surface CR1 level was quantified by flow cytometry on formaldehyde-fixed RBC samples, as described in detail previously.^66^ Previous data indicated that CR1 levels determined on formaldehyde-fixed RBCs are equivalent to those determined using fresh RBCs, as long as the standards (with known CR1 levels) are fixed and stored in an identical way.^66^ Fixed cells were stored at 4 **°**C and CR1 quantified within 8 weeks. Briefly, fixed RBC pellets were stained in duplicate with 0.5 μg ml^−1^ of mouse anti-CR1 monoclonal antibody J3D3 (catalogue number COIM0195, Beckman-Coulter Inc., Fullerton, CA, USA) followed by a secondary staining with 5 μg ml^−1^ of Alexa Fluor 488-conjugated goat anti-mouse IgG (catalogue number A-11001, Molecular Probes, Leiden, the Netherlands) and the mean fluorescence intensity determined on a FC500 flow cytometer (Beckman-Coulter Inc.). CR1 levels of unknown samples were then extrapolated from a standard curve generated using three RBC samples of known CR1 levels (low ~350, intermediate ~650 and high CR1 levels ~900) included in each experiment, and prepared and stored in the same way as the test samples.^66^ Thirteen samples were collected but were not included in the study due aberrant forward/side scatter plots or readings that were >2 standard deviations above the highest point on the standard curve.

### DNA extraction and genotyping

DNA was extracted either from fresh whole blood samples using the semi-automated ABI PRISM 6100 Nucleic acid prep station (Applied Biosystems, Foster City, CA, USA) or from EDTA blood samples, previously stored at −80 **°**C, using the QIAamp DNA and Blood Mini Kit (Qiagen, Valencia, CA, USA). Genotyping for the *Sl* and *McC* blood group genotypes was carried out as described previously using the SEQUENOM MassARRAY platform (Agena Biosciences, Hamburg, Germany) multiplex system following DNA amplification by whole-genome amplification.^67^ ABO blood groups were determined by either standard haemagglutination methods or were inferred from A, B and O SNP haplotypes determine by Sequenom typing as detailed previously.^67^ Genotyping for HbAS and α^+^thalassaemia was by PCR as described in detail elsewhere.^68, 69^

### MCV determination

Full blood counts for the determination of RBC MCV were performed on fresh EDTA blood samples from the mild disease cohort using a Coulter MDII-18 machine (Beckman-Coulter Inc.).

### Sample availability

A total of 3535 samples were successfully typed for RBC CR1 level, HbS and α^+^thalassaemia genotypes, whereas ABO and the *Sl* and *McC* Knops genotypes were available for all samples except the 460 samples from the Birth Cohort Study 2. MCV data were only available for the 147 samples collected from members of the Mild Disease Cohort Study.

### Statistical analysis

Statistical analyses were conducted using STATA v11 (StataCorp LP, TX, USA) and Graph Pad Prism v5 (GraphPad Software Inc, San Diego, CA, USA) and all graphs were generated using GraphPad Prism. The relationship between RBC CR1 level and age as a categorical variable was assessed using one-way analysis of variance with Tukey's *post-hoc* test to correct for multiple comparisons. We also analysed the relationship between RBC CR1 level and age as a continuous variable using piecewise-linear regression analysis allowing for adjustment for confounding by α^+^thalassaemia and *HbS*. Unlike conventional linear models that assume linearity over the whole range of a continuous independent variable, this model assumes a linear effect within defined ranges of the continuous variable that are connected. The age categories and ranges chosen were 0–3 months, 4–6 months, 7–12 months, 13–24 months, >2–4 years, >4–8 years and >8–13 years, as used in previous studies.^16, 29^ The relationship between RBC CR1 level and α^+^thalassaemia, *HbS*, ABO blood group and the *Sl* and *McC* genotypes was tested by multivariate linear regression analysis. Each variable was first tested in a univariate regression model and all explanatory variables displaying a *P* value of <0.05 (all except ABO) were included in a final multivariate model. Age as a categorical variable was also included in the multivariate model, using the age intervals described above. ABO blood group and *Sl* and *McC* genotypes were not available for the 460 children from Birth Cohort Study 2, therefore the multivariate analyses are restricted to the 3075 samples with full genotype data. For ease of interpretation, predicted mean CR1 levels and 95% CIs estimated from the multivariate regression results are presented in figures, rather than regression coefficients, using the ‘margins' post-estimation command in Stata. A two-sided significance level of <0.05 was considered statistically significant for all analyses.

### Ethical statement

Individual informed written consent to collect blood was obtained from all participants' parents or legal guardians. The study was approved by the Kenya Medical Research Institute National Ethical Review Committee in Nairobi and was conducted in accordance with the Declaration of Helsinki.

## Figures and Tables

**Table 1:** Regression coefficients for the relationship between age and RBC CR1 level over different age ranges in Kilifi, Kenya

*Age intervals*	*Regression coefficients*	*95% CI*	P *value*	N
0–3 Months	−31.24	−64.83–2.35	0.068	73
4–6 Months	−48.08	−62.08–−34.07	<0.001	877
7–12 Months	−6.22	−9.65–−2.79	<0.001	1877
13–24 Months	−4.13	−7.16–−1.09	0.008	547
>2–4 Years	4.25	0.73–7.78	0.018	42
>4–8 Years	−0.33	−2.66–1.99	0.778	62
>8–13 Years	0.94	−1.31–3.19	0.411	57

Abbreviation: CI, confidence interval.

Differences in RBC CR1 levels by age as a continuous variable were analysed by piecewise-linear regression analysis with adjustment for confounding by α^+^thalassaemia and *HbS*. Age was classified into seven different ranges represented as 0–3 months, 4–6 months, 7–12 months, 1–2 years, >2–4 years, >4–8 years and >8–13 years.
