# Supplementary figures and images for: Red blood cell complement receptor one level varies with Knops blood group, α+thalassaemia and age among Kenyan children
Source: Genes Immun. 2016 Feb 4;17(3):171–8. doi: 10.1038/gene.2016.2 (PMC4842007; doi:10.1038/gene.2016.2)

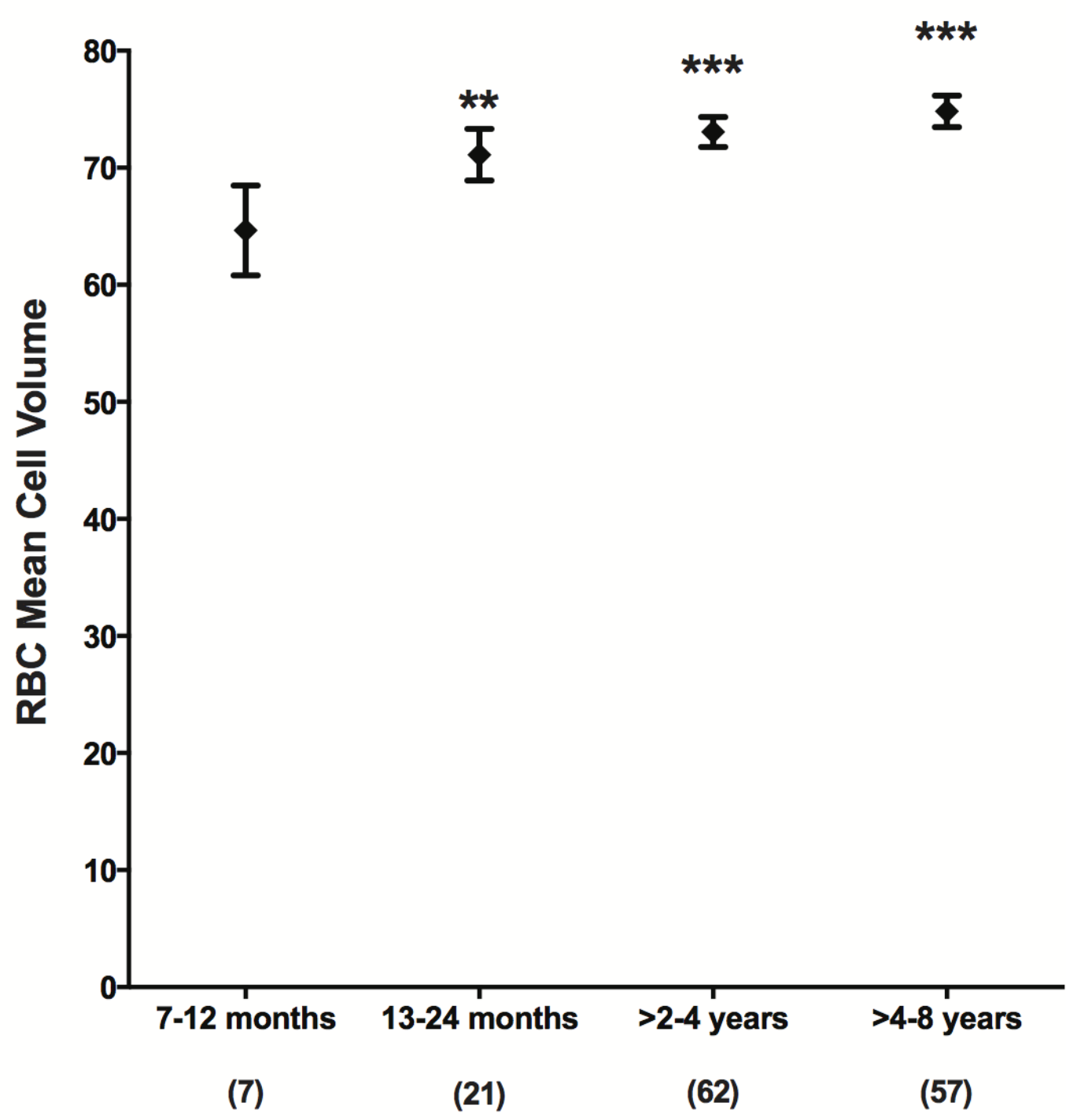

Supplement: Supplementary Figure 1 [file gene20162x1.pdf]
